# The Transcription Factor C/EBPβ Promotes HFL-1 Cell Migration, Proliferation, and Inflammation by Activating lncRNA HAS2-AS1 in Hypoxia

**DOI:** 10.3389/fcell.2021.651913

**Published:** 2021-03-12

**Authors:** Xue Yang, Fei Qi, Shanchen Wei, Lianjun Lin, Xinmin Liu

**Affiliations:** Department of Geriatrics, Peking University First Hospital, Beijing, China

**Keywords:** hypoxic pulmonary hypertension, HAS2-AS1, C/EBPβ, inflammation, HFL-1 cells

## Abstract

**Objective:**

Recent studies were widely concerned about the role of lncRNAs in hypoxic pulmonary hypertension (HPH). HAS2 was found significantly highly expressed in HPH, but the antisense of HAS2 (HAS2-AS1) has not been explored in HPH, providing a new potential therapeutic target of HPH.

**Methods:**

In this study, human fetal lung fibroblast-1 (HFL-1) cells were cultured under hypoxia conditions to stimulate the pathological process of HPH. Transwell and wound-healing assays were used to detect HFL-1 cell migration, and CCK 8 assay was used to detect cell proliferation. The upstream transcription factor of HAS2-AS1 was predicted by JASPAR website, and the binding site between C/EBPβ and HAS2-AS1 was predicted by JASPAR, too. In order to verify the association between C/EBPβ and the HAS2 promoter region, we used chromatin immunoprecipitation (ChIP) and dual luciferase reporter gene detection, western blot to detect the expression of inflammation-related proteins, and qRT-PCR to detect the expression of HAS2-AS1 and HAS2. Idiopathic pulmonary fibrosis (IPF) with HPH patient microarray data was downloaded from the GEO database and analyzed by R software.

**Results:**

Our study showed that HAS2-AS1 and C/EBPβ were highly expressed in hypoxic HFL-1 cells, and the knockdown of HAS2-AS1 expression could inhibit the proliferation, migration, and inflammatory response of HFL-1 cells. C/EBPβ binds to the promoter region of HAS2-AS1 and has a positive regulation effect on the transcription of HAS2-AS1. Furthermore, C/EBPβ can regulate the proliferation, migration, and inflammatory response of HFL-1 cells through HAS2-AS1.

**Conclusion:**

This study suggested that C/EBPβ could upregulate HAS2-AS1 expression and induce HFL-1 cell proliferation, migration, and inflammation response.

## Introduction

Hypoxic pulmonary hypertension (HPH) is mainly caused by enhanced pulmonary vasoconstriction and abnormal proliferation of smooth muscle cells in the middle layer of terminal pulmonary arteriole. HPH is often secondary to the chronic obstructive pulmonary hypertension (COPD), idiopathic pulmonary fibrosis (IPF), obstructive sleep apnea-hypopnea syndrome (OSAHS), and so on (Seeger et al., 2013; Galie et al., 2016). HPH is an irreversible and refractory disease, the pathophysiology of HPH including vascular endothelial cell and smooth muscle cell (PASMC) proliferation, adventitia fibroblasts proliferation, and migration, leading to vascular remodeling (Tuder, 2017). In recent studies, adventitial fibroblasts, rather than endothelia or smooth muscle cells, are considered the principal sensor tissue in response to oxidative stress (Wang et al., 2010). In addition, the development of HPH deals profoundly with inflammation and multiple cytokines (Stenmark et al., 2006). The cytokines including TNF-α, IL-6, and IL-1β could accelerate vascular remodeling (Rabinovitch et al., 2014) and aggravated the severity of PAH and associated with worse outcomes (Hurst et al., 2017; Tamura et al., 2018; Trankle et al., 2019). Hypoxia could activate fibroblasts through epigenetic mechanisms and increase the infiltration of cytokines, chemokines, and extracellular matrix, which regulate the function and structure of the whole lung tissues (El Kasmi et al., 2014; Wang et al., 2014).

Upcoming studies have revealed long non-coding RNA (lncRNA) is a novel gene deficiency of HPH progression. For example, lncRNA MALAT1 is a star gene significantly elevated in hypoxia PASMCs (Wang et al., 2019) and knockdown of lncRNA MEG3 affected the proliferation and migration of PASMCs *in vitro* through the p53 pathway (Sun et al., 2017). However, these studies mainly focused on PASMCs, while the regulatory mechanism of lncRNA in HPH remains unclear. HAS2-AS1 as a lncRNA, which is believed to be an oncogene in glioblastoma (Zhang et al., 2019), oral squamous cell carcinoma (Zhu et al., 2017), and epithelial ovarian cancer (Tong et al., 2019).

In fact, HAS2-AS1, as the natural antisense RNA of HAS2, could promote the expression of HAS2 by forming the heterodimer of HAS2 mRNA/HAS2-AS1 (Michael et al., 2011). HAS2 is a main component of most extracellular matrix and is associated with disease progression of experimental PAH, and inhibition of HAS2 can alleviate PAH and pulmonary fibrosis-related diseases (Collum et al., 2017). Nevertheless, the expression and function of HAS2-AS1 in HPH associated with IPF, including hypoxic fibroblasts, have not been reported yet.

Transcription factors can bind to lncRNA promoters and regulate gene expression, including HAS2-AS1 (Wang J. T. et al., 2020). CCAAT enhancer-binding protein (C/EBPβ) is a key transcription factor that binds to lncRNA LEF1-AS1 to regulate the proliferation and invasion of HCC cells (Gao et al., 2020). In our study, we predicted a binding relationship between C/EBPβ and the promoter region of HAS2-AS1 using bioinformatic analysis and explore the influence on proliferation, migration, and inflammation of human fetal lung fibroblast-1 (HFL-1) cells, which provided a potential novel target for the treatment of HPH associated with IPF.

## Materials and Methods

### Cell Culture and Hypoxia Treatment

Human fetal lung fibroblast-1 (Type Culture Collection Center, Chinese Academy of Sciences) were cultured in Ham’s F-12K medium containing 10% fetal bovine serum (Gibco, Thermo Fisher Scientific), 100 mg/ml streptomycin and 100 U/ml penicillin in a wet atmosphere of 5% CO_2_. Cells were equalized with N_2_ under 1% O_2_ (hypoxia) and 21% O_2_ (normoxia) conditions and exposed to hypoxia in a three-gas incubator (Don Whitley, England).

### Cell Transfection and Stable Cell Establishment

Si-HAS2-AS1, si-C/EBPβ, and their negative control were synthesized by ABM (Jiangsu, China), and HFL-1 cells were transfected according to the instructions by Lipofectamine 2000 (Thermo Fisher Scientific, Inc.). The HAS2-AS1 overexpressed lentivirus (Hanheng, China) was transfected into cells with 4 μg/ml polybrene (Hanheng, China), and after 48 h infection, stable clones were screened out using 2 μg/ml puromycin. The siRNA sequences are shown in Table 1.

**TABLE 1:** The sequences of siRNAs.

Name	Sequences
Si-HAS2-AS1-1	5′-GCGGUGUCCUUGAGUCCAATT-3′
Si-HAS2-AS1-2	5′-CCUCAUACAGACCCUCCUUTT-3′
Si-HAS2-AS1-3	5′-GGAACUGCCGUGACGAAUUTT-3′
Si-C/EBPβ-1	5′-CCAUGGAAGUGGCCAACUUTT-3′
Si-C/EBPβ-2	5′-GCAACCCACGUGUAACUGUTT-3′
Si-C/EBPβ-3	5′-GUGUACAGAUGAAUGAUAATT-3′
Si-NC	5′-CACCUCUAGUUGUCCCUGATT-3′

### Grouping Steps

After transfecting with si-HAS2-AS1 and negative control, HFL-1 cells were divided into four groups to detect the effect of HAS2-AS1: si-NC under normoxia and hypoxia and si-HAS2-AS1 under normoxia and hypoxia. To explore the regulation of C/EBPβ on HAS2-AS1, and the changes of inflammatory phenotypes, we set up four groups for analysis after overexpressing HAS2-AS1 and knocking down C/EBPβ: si-NC + oe-NC, si-C/EBPβ + oe-NC, si-C/EBPβ + oe-HAS2-AS1, and si-NC + oe-HAS2-AS1.

### RNA Extraction and Quantitative Real-Time PCR

The 7500 real-time PCR system of American Applied Biosystems was used for quantitative real-time PCR (qRT-PCR) detection. Total RNA of HFL-1 cells was extracted using Trizol reagent (Invitrogen), and reverse transcription was performed using reverse transcriptase (Thermo Fisher Scientific, Inc.). The primers were obtained from Sangon Biotech Co. (Shanghai, China) as listed in Supplementary Table 1. After normalization with β-actin, the relative expressions of HAS2-AS1, HAS2, and C/EBPβ were compared by 2^–ΔΔ*CT*^ method.

### Cell Counting Kit-8

Cell Counting Kit-8 (CCK8, Dojindo, Japan) assay was used to detect the proliferative abilities of HFL-1 cells. HFL-1 cells (5 × 10^3^ cells/well) were inoculated into 96-well plate and cultured for 24, 48, and 72 h under hypoxic and normoxic conditions, respectively, meanwhile, transfected cells were tested at the same time points. Each well was incubated with 10 μl CCK8 solution for 1 h, and the absorbance was measured at 450 nm wavelength.

### Wound-Healing Assay

When HFL-1 cells grew about 80% in confluence, a pipette tip was used to create a scratch. The cells were gently washed twice with PBS and cultured with fresh medium for 24 h under hypoxia and normoxia. The cell images were collected under inverted optical microscope, and the number of migratory cells was counted and the migration ability was evaluated.

### Transwell Assay

Approximately 3 × 10^4^ HFL-1 cells (200 μl) were inoculated into the upper Transwell chamber (Corning, United States), and 750 μl Ham’s F-12K with 10% FBS was added to the lower cavity. After 24 h of culture, 4% paraformaldehyde and 0.4% crystal violet were stained, and substrate membrane was fixed. The images were observed using a microscope and selected randomly, and the number of cells were counted by ImageJ software.

### Chromatin Immunoprecipitation

To detect C/EBPβ and HAS2-AS1-binding sites, ChIP Assay Kit (ABCAM) was used to perform chromatin immunoprecipitation (ChIP) according to the instructions and established protocol (Ke et al., 2020); 200–1,000 bp DNA fragments were obtained by sonication. HFL-1 cells were cross-linked with formaldehyde. Subsequently, non-specific IgG antibodies (ab172730, Abcam) and C/EBPβ antibody (ab32358, Abcam) were precipitated with chromatin overnight at 4°C. NaCl was used to reverse DNA cross-linking, qRT-PCR was used to detect HAS2-AS1 level, and 2^–ΔΔ*CT*^ was used to calculate.

### Luciferase Assay

The C/EBPβ binding site of HAS2-AS1 promoter was cloned into firefly luciferase reporter vector pGL3(Promega, Madison, WI, United States) using *Kpn*l and *Xho*I restriction sites. Then, the C/EBPβ construct expression vector or the empty vector was cotransfected with the wild or mutant vector, and Lipofectamine 2000 (Invitrogen) was transfected according to the instructions. After 48 h, relative luciferase activity was measured using a Dual-Luciferase^®^ Reporter Assay System (E1910, Promega).

### Western Blot

The total protein was extracted with RIPA lysate, and the concentration was determined with BCA kit (Beyotime, Shanghai, China). Twenty micrograms of protein per lane was isolated by 10 or 12% SDS-PAGE and transferred to PVDF membrane (Millipore, United States). After the film was sealed, incubate with 5% skim milk powder and primary antibody at 4°C overnight. HRP-conjugated secondary antibodies were washed three times with TBST and cultured in membrane at room temperature for 1 h. Finally, enhanced chemiluminescence western blotting detection system was used to observe the protein bands and the relative protein expression was calculated by Image J software. In our experiments, the antibodies including HAS2 (dilution 1:100, Sc-514737, Santa Cruz), C/EBPβ (dilution 1:200, Sc-7962, Santa Cruz), TNF-α (dilution 1:200, Sc-28318, Santa Cruz), IL-6 (dilution 1:1,000, 66146-1-Ig, Proteintech), IL-1β (dilution 1:1,000, #12242, CST), β-actin (dilution 1:2000, 60008-1-Ig, Proteintech), and goat anti-mouse IgG (dilution 1:5,000, ZSGB-Bio, China).

### Microarray Data Collection and Analysis

GSE24988 was acquired from a comprehensive gene expression database^1^ of gene expression profile datasets, including 116 cases of patients with lung tissue, among them, 22 IPFs with no pH (MPAP ≤ 20 mmHg), 45 IPFs with moderate pH (MPAP 21–39 mmHg), 17 IPFs with severe pH (MPAP ≥ 40 mmHg), and 32 PH validation. A robust multiarray averaging algorithm of the affy package of R software is used to transform the original data of CEL files into expression data. Each probe matched with related genes through Affymetrix Human Gene 1.0 ST Array platform by using the biconductor annotation function of R software. The average expression value of multiple probes for a given gene was obtained.

### Statistical Analysis

SPSS 19.0 software was used for statistical analysis, and GraphPad Prism 6 was used to make charts. Student’s *t*-test was used for comparison of two groups. One-way analysis of variance (ANOVA) was used for comparison of multiple groups. All the results were expressed as mean ± standard deviation and repeated three times independently, *P* < 0.05 was considered statistically significant.

## Results

### Hypoxia Promotes HFL-1 Proliferation, Migration, and HAS2-AS1 Expression

**FIGURE 1:**
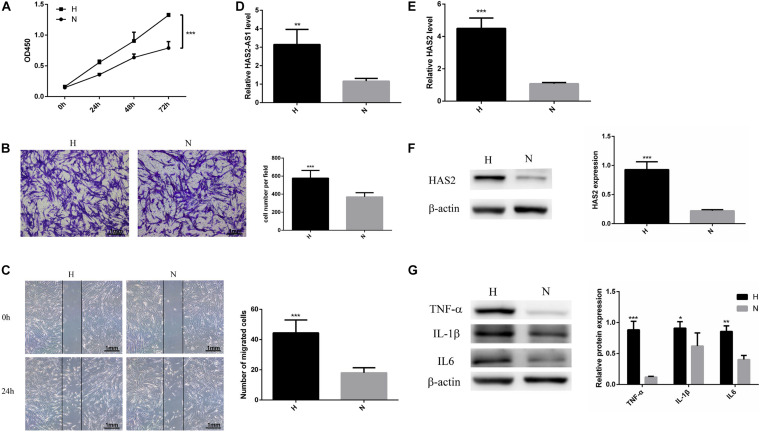
Hypoxia promotes HFL-1 proliferation, migration, and HAS2-AS1 expression. **(A)** HFL-1 cell proliferation increased in hypoxia at 24–72 h. **(B,C)** Transwell assay and wound-healing assay showed that the migration of HFL-1 cells was enhanced under hypoxia. **(D)** The expression of HAS2-AS1 in HFL-1 cells was detected by qRT-PCR. **(E,F)** The expression of HAS2 of HFL-1 cells was detected by qRT-PCR and western blot. **(G)** The expressions of inflammation-related cytokines including TNF-α, IL-6, and IL-1β were detected by western blot. H, hypoxia; N, normoxia (*P < 0.05; **P < 0.01; ***P < 0.001).

To determine the effects of hypoxic conditions on the HFL-1, the cell proliferation and migration were detected. The growth curves showed that hypoxic conditions promote HFL-1 viability at 24–72 h (*P* < 0.05, Figure 1A). By using wound-healing and Transwell migration assays, the effect of hypoxia on cell migration was measured. Compared with normoxic group, as shown in Figures 1B,C, the HFL-1 in hypoxia showed stronger migration abilities.

Multiple studies have reported that HAS2 has an important role in pulmonary hypertension (Ormiston et al., 2010; Collum et al., 2017), but there is no one reported HAS2-AS1 expression in pulmonary hypertension. Here, we chose HAS2-AS1 for our experiments. Our results revealed that the expression of HAS2-AS1 was significantly increased in hypoxic exposure (Figure 1D). Furthermore, HAS2 expression was dramatically upregulated at mRNA and protein levels (Figures 1E,F). Besides, considering the close relationship between hypoxia and inflammation, we performed the expression of TNF-α, IL-6, and IL-1β and found that their expression levels all increased in hypoxia (Figure 1G). These results indicated that hypoxia could activate HFL-1 cells and increase the HAS2-AS1 expression and inflammation expression.

### Knockdown HAS2-AS1 Inhibits HFL-1 Proliferation, Migration, and Inflammation

**FIGURE 2:**
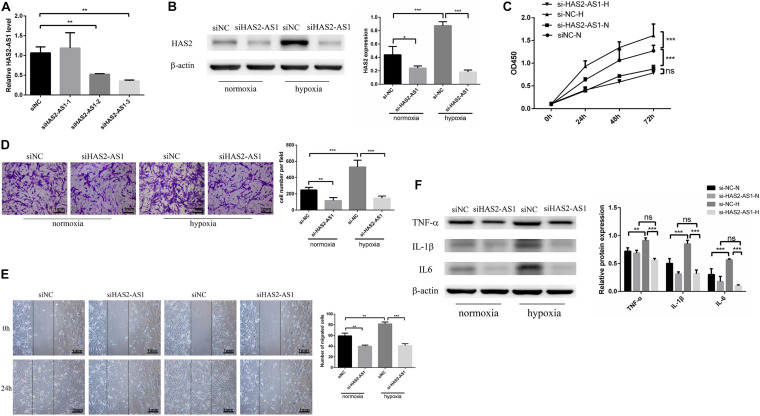
Knockdown HAS2-AS1 inhibits HFL-1 proliferation, migration and inflammation. **(A)** qRT-PCR was used to select the interference efficiency of three siRNAs (siHAS2-AS1-1, siHAS2-AS1-2, siHAS2-AS1-3). **(B)** HAS2 expression after knocking down HAS2-AS1 was tested by western blot. **(C)** CCK8 assay was used to detect the proliferation of HFL-1 cells after knocking down HAS2-AS1 under hypoxia and normoxia. **(D,E)** The migration ability of HAS2-AS1 knocked-down HFL-1 cells was detected by Transwell and wound-healing assays. **(F)** TNF-α, IL-6, and IL-1β expressions were detected by western blot in silencing HAS2-AS1 and silencing control groups in hypoxia and normoxia. si-NC-N, si-NC + normoxia; si-NC-H, si-NC + hypoxia; si-HAS2-AS1-N, si-HAS2-AS1 + normoxia; si-HAS2-AS1-H, si-HAS2-AS1 + hypoxia (*P < 0.05; **P < 0.01; ***P < 0.001).

We used siRNA to interfered the HAS2-AS1 expression in HFL-1 cells to verify whether HAS2-AS1 was a key point to promoting cell phenotypic changes. We selected siHAS2-AS1-3 for the following experiments based on the highest silencing efficiency (Figure 2A). The HAS2 expression was downregulated following HAS2-AS1 knockdown (Figure 2B). The result of CCK8 (Figure 2C) revealed that HAS2-AS1 knockout could inhibit cell proliferation under normoxia and hypoxia. Subsequently, cell migration ability was measured by Transwell (Figure 2D) and wound-healing assays (Figure 2E). The result showed that siHAS2-AS1 considerably weakened the migration ability compared with siNC group under normoxia and hypoxia. In addition, we also tested the inflammation expression after transfecting si-HAS2-AS1 and found that the level of TNF-α, IL-6, and IL-1β were decreased, especially under hypoxia (Figure 2F). Hence, based on these results, HAS2-AS1 could be considered a novel key to promote HFL-1 proliferation and migration in hypoxia.

### Transcription Factor C/EBPβ Regulates HAS2-AS1 Expression by Binding to Its Promoter Region

**FIGURE 3:**
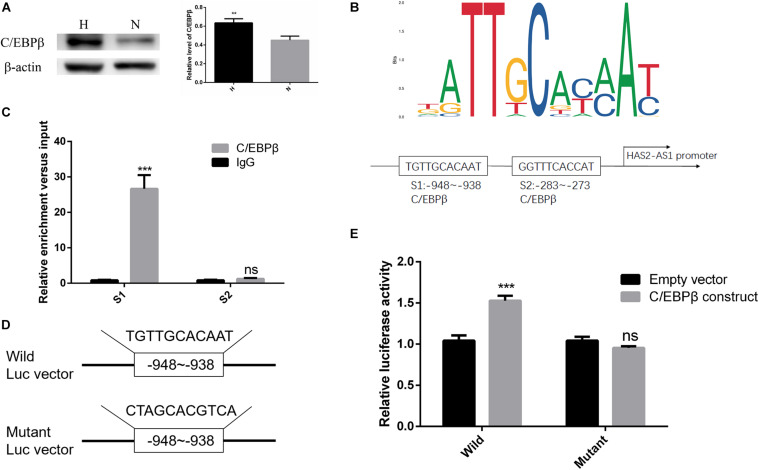
Transcription factor C/EBPβ-adjusted HAS2-AS1 expression by binding to its promoter region. **(A)** The expression of C/EBPβ was increased in HFL-1 cells under hypoxia. **(B)** JASPAR website was used to predict the binding sites of C/EBPβ in HAS2-AS1 promoter region, and the first three binding sequences were selected out. **(C)** The predicted binding sequences of C/EBPβ on HAS2-AS1 promoter region were detected by CHIP method. **(D)** Wild- and mutant-type vectors were constructed according to the CHIP result. **(E)** Dual-Luciferase reporter assay was used to verify the binding sequence (**P < 0.01; ***P < 0.001).

Currently, many studies showed that transcription factors can regulate the expression of lncRNA by binding to the upstream promoter region (Wang J. et al., 2020; Zhang et al., 2020). Therefore, we used JASPAR database^2^ to predict possible transcription factors and obtained high score choice C/EBPβ. Studies showed that C/EBPβ is a crucial factor in hypoxia-induced inflammation (Yu et al., 2019; Fujii et al., 2020), and our result showed C/EBPβ was upregulated in hypoxia (Figure 3A). However, whether it can regulate HAS2-AS1 and the mechanism remains unknown. According to the binding sites predicted by JASPAR website, the first two binding sequences of HAS2-AS1 promoter region C/EBP were selected (Figure 3B). To verify the binding relationship between C/EBPβ and HAS2-AS1 promoter region, we performed ChIP analysis. Interestingly, S1 was the only binding site of C/EBPβ in the HAS2-AS1 promoter region, indicating that –938 to –948 was the binding region (Figure 3C). For further verification, wild-type and mutant-type vectors (Figure 3D) were constructed and cotransfected with the reporter vector. Luciferase activity was detected, and the result showed that the mutation of C/EBPβ resulted in a dramatic decrease in luciferase activity (Figure 3E). Overall, these data suggested that the increased expression of HAS2-AS1 of HFL-1 cells in hypoxia may be partly caused by the activation of C/EBPβ.

### Knockdown C/EBPβ Suppresses HFL-1 Proliferation, Migration, and Inflammation by Inhibiting HAS2-AS1

**FIGURE 4:**
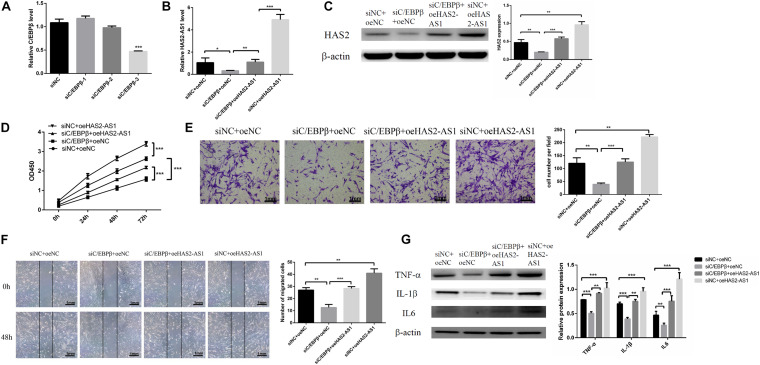
Knockdown C/EBPβ suppresses HFL-1 proliferation, migration, and inflammation by inhibiting HAS2-AS1. **(A)** qRT-PCR was used to verify the interference efficiency of C/EBPβ. **(B,C)** The expressions of HAS2-AS1 and HAS2 were detected using qRT-PCR and western blot. **(D)** The proliferation of HFL-1 cells was decreased after knocking down C/EBPβ and overexpressing HAS2-AS1. **(E,F)** The migration of HFL-1 cells after silencing C/EBPβ and overexpression of HAS2-AS1 was detected by Transwell and wound-healing assays. **(G)** The expressions of inflammation-related proteins TNF-α, IL-6, and IL-1β in each group were detected by western blot. si-NC, small interference negative control; oeNC, overexpressed negative control (*P < 0.05; **P < 0.01; ***P < 0.001).

We knocked down the expression of C/EBPβ with siRNA (Figure 4A) and overexpressed HAS2-AS1 with lentivirus to confirm that C/EBPβ influences HFL-1 cell proliferation, migration, and inflammation by inhibiting HAS2-AS1. Firstly, we detected the expression of HAS2-AS1 by qRT-PCR. The expression level of HAS2-AS1 and HAS2 was decreased due to the downregulation of C/EBPβ, which are shown in Figures 4B,C. However, the result was reversed by the HAS2-AS1 overexpression. Afterward, the proliferation experiment was performed by CCK8 assay. The result revealed that interfering the expression of C/EBPβ could inhibit cell proliferation and HAS2-AS1 overexpression could restore viability (Figure 4D). Similarly, knocking down the expression of C/EBPβ suppressed HFL-1 migration and overexpressing HAS2-AS1 could repair the migration ability to some extent (Figures 4E,F). Finally, inflammation-related protein, TNF-α, IL-6, and IL-1β were detected by western blot. Knockdown C/EBPβ inhibited TNF-α, IL-6, and IL-1β expression compared with siNC group, but these inflammation proteins were upregulated again as a result of HAS2-AS1 overexpression (Figure 4G). The results concluded that knockdown C/EBPβ suppressed the expression of HAS2-AS1, thus inhibiting HFL-1 proliferation, migration, and inflammation.

### C/EBPβ and HAS2 Were Increased in IPF With Severe PH Patients

**FIGURE 5:**
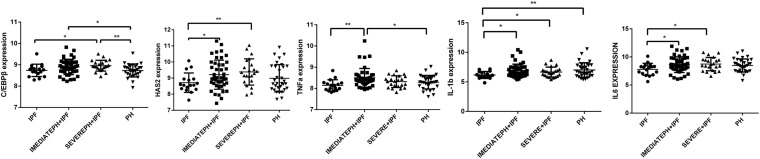
C/EBPβ and HAS2 were increased in IPF with severe PH patients. One-way analysis of variance (ANOVA) was used to analyze gene expression in GSE24988 dataset. IPF, idiopathic pulmonary fibrosis; intermediate PH with IPF, mPAP 21-39mmHg IPF patients; severe PH with IPF, mPAP ≥ 40mmHg IPF patients; PH, pulmonary hypertension (*P < 0.05; **P < 0.01).

Through download and analysis of the microassay dataset, we found that the expression of C/EBPβ and HAS2 were evaluated in IPF with severe PH patients compared with pure IPF patients (Figure 5). What is more, the expressions of TNF-α, IL-6, and IL-1β were also highly expressed in IPF with severe PH group. These results obtained from the patients’ lung tissues supported the findings in our cellular experiments.

## Discussion

More and more studies have been exploring the lncRNAs in HPH; the functions and mechanisms are the hot topics which researchers cared about the most. HAS2-AS1 is a natural antisense gene of HAS2. However, the expression of HAS2-AS1 in HPH and its function have not been studied. Our study showed that the HAS2 was significantly increased in patients with PAH and inhibition of HAS2 could alleviate PAH in experimental rat models (Collum et al., 2017). In lung tissues of HPH patients caused by COPD, HAS2 level was positively correlated with pulmonary arterial pressure (Karmouty-Quintana et al., 2013). Some researchers found that endothelin-1 could induce HAS2 expression in PASMCs (Yeager et al., 2012), and this function required the production of cytokines including IL-6 and TNF-α (Vigetti et al., 2010). On account of the limited number of tissues available for HPH, the lncRNA expression of human lung tissue have not been detected. Hence, HFL-1 cells, which were considered functional cells in pulmonary hypertension associated with IPF, were selected as our research object. We found that the proliferation and migration ability of HFL-1 cells were significantly enhanced under hypoxia condition, and the expression of HAS2-AS1 and HAS2 was detected under hypoxia condition. What is more, the cytokines related to inflammation including TNF-α, IL-1β, and IL-6 were highly expressed in hypoxia. These results suggested that hypoxic condition activates the changes in the phenotype of HFL-1 cells.

For further study, we knocked down HAS2-AS1 in HFL-1 cells under hypoxia and normoxia conditions. Our results demonstrated that the migration and proliferation of HFL-1 cells under hypoxia were dramatically inhibited by silencing HAS2-AS1, and HAS2 expression was downregulated. Interestingly, the expression of cytokines was also decreased. Previous studies on HAS2-AS1 did not involve the expression of inflammation. Our study fully proved that HAS2-AS1 knockout could inhibit the proliferation, migration, and inflammatory response of HFL-1 cells, suggesting that HAS2-AS1 may be a potential target of HPH.

Previous researches have suggested that HAS2-AS1 is regulated by transcription factors such as USF-1 (Wang J. T. et al., 2020), CREB1 (Tong et al., 2019), and HIF-1α (Zhu et al., 2017). We need to further investigate whether the effect of HAS2-AS1 on inflammation expression is also regulated by transcription factors. We then used multiple databases to filter for transcription factors that might bind to HAS2-AS1 promoter region and found C/EBPβ finally. C/EBPβ, a member of the CCAAT transcription enhancer family, has shown playing a role in promoting hypoxia-induced inflammation (Gao et al., 2020). C/EBPβ was highly expressed in hypoxic vascular endothelial cells (Feng et al., 2019) but lowly expressed in hypoxic-induced fibroblast-like synoviocytes (Yu et al., 2019). Therefore, the expression of C/EBPβ may be different in different cell types. In our study, we showed that C/EBPβ was highly expressed during hypoxia. To verify our hypothesis, we used ChIP and luciferase assay to verify the binding of C/EBPβ to the HAS2-AS1 promoter region. Downregulating the expression of C/EBPβ, the HAS2-AS1 expression was decreased and the proliferation and migration abilities were weakened. In addition, the expression of TNF-α, IL-1β, and IL-6 were reduced. However, these inhibitory effects of C/EBPβ could be rescued by overexpression of HAS2-AS1.

Furthermore, using gene expression microarray data from the GEO database, we found that compared with simple IPF patients, HAS2 and C/EBPβ expression increased in patients with PH, especially in patients with severe PH (MPAP ≥ 40 mmHg). Otherwise, the cytokines including TNF-α, IL-1β, and IL-6 were evaluated in IPF with severe PH group. Although we were unable to directly measure HAS2-AS1 expression in human HPH lung tissue, our study demonstrated a novel mechanism by which C/EBPβ regulates inflammation by binding to the promoter region of HAS2-AS1.

In the past, deposition of extracellular matrix (Thenappan et al., 2018) and inflammation infiltration (Tuder et al., 2013) were considered very important pathologic processes of HPH, but the relationship between these two processes have not been demonstrated clearly. In summary, our study confirmed that hypoxic condition could promote the expression of C/EBPβ (a transcription factor related to inflammation) and HAS2-AS1 (a lncRNA related to extracellular matrix), and C/EBPβ could promote cell migration, proliferation, and inflammatory response through binding to the promoter region of HAS2-AS1 and activate its transcription. This revealed a new mechanism by which C/EBPβ influenced the inflammatory response under hypoxia conditions and provides a potential therapeutic target for HPH, especially HPH with IPF.

## Data Availability Statement

The original contributions presented in the study are included in the article/Supplementary Material, further inquiries can be directed to the corresponding author/s.

## Author Contributions

XY, LL, and XL: study concept and design, drafting of the manuscript, and study supervision. XY, FQ, and SW: analysis and interpretation of data. FQ and SW: administrative, technical, and material support. All authors have read and approved the manuscript.

## Conflict of Interest

The authors declare that the research was conducted in the absence of any commercial or financial relationships that could be construed as a potential conflict of interest.

## Footnotes

1 https://www.ncbi.nlm.nih.gov/geo/

2 http://jaspar.genereg.net/
